# Supplementary material for: Transmission phase read-out of a large quantum dot in a nanowire interferometer
Source: Nat Commun. 2020 Jul 22;11:3666. doi: 10.1038/s41467-020-17461-5 (PMC7376064; doi:10.1038/s41467-020-17461-5)
Supplement: Supplementary file 1 — Supplementary Information [file 41467_2020_17461_MOESM1_ESM.pdf]

Supplementary Information

Transmission phase read-out of a large quantum dot in a nanowire interferometer

F. Borsoi *et al.*

Supplementary Note 1. Transfer of nanowire network devices

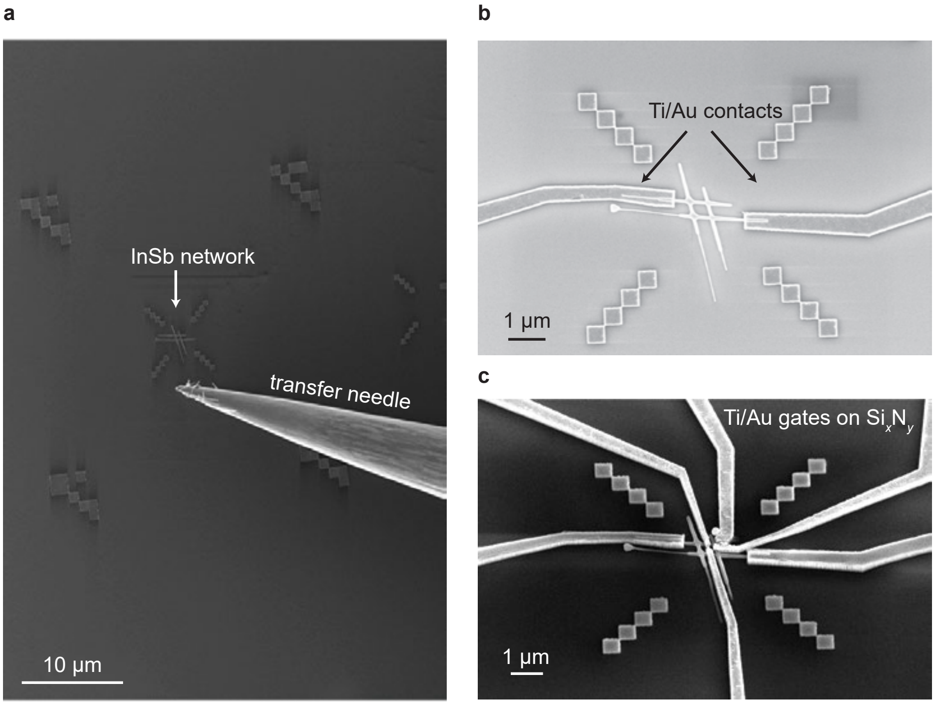

Supplementary Figure 1: Transfer of the InSb nanowire networks. **a** Mechanical transfer of a nanowire network with a nanomanipulator in a scanning electron microscope. The device is placed in the vicinity of pre-patterned alignment markers. **b**, **c** Scanning electron micrographs of the device after the fabrication of the Ti/Au contacts and the Ti/Au top gates that are deposited onto a dielectric layer of Si_xN_y, respectively.

Supplementary Note 2. Back-gate dependence

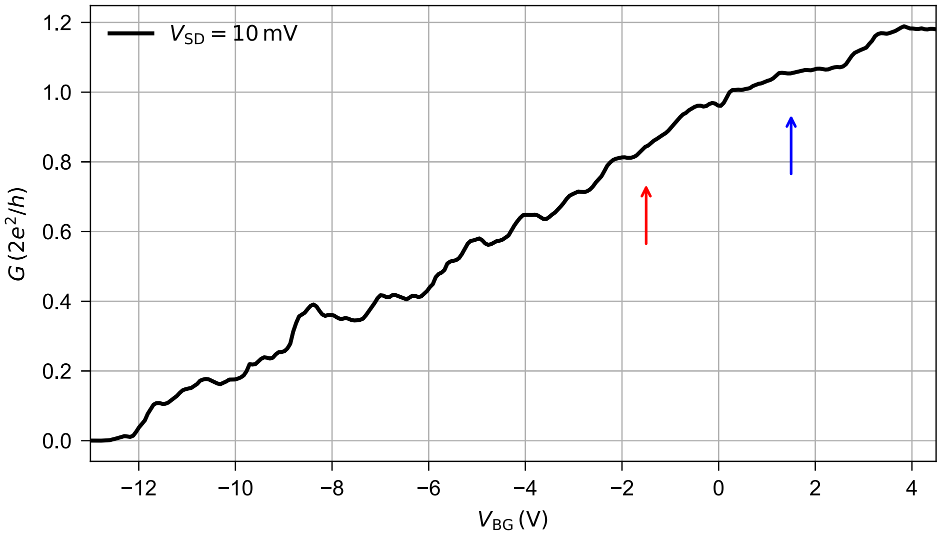

Supplementary Figure 2: G vs. V_{BG} at 10 mV bias voltage. The conductance increases linearly with respect to the global back gate in agreement with the Drude model. The blue and red arrows at 1.5 V and −1.5 V, respectively, indicate the two working points at which the data discussed in the main text are taken. In the second case, the electron density (roughly proportional to the conductance) is only $\sim 20\%$ lower than in the first case. Therefore, we can conclude that the quantum dot is in both cases in the many-electron regime.

Supplementary Note 3. Fourier spectrum of the magneto-conductance oscillations

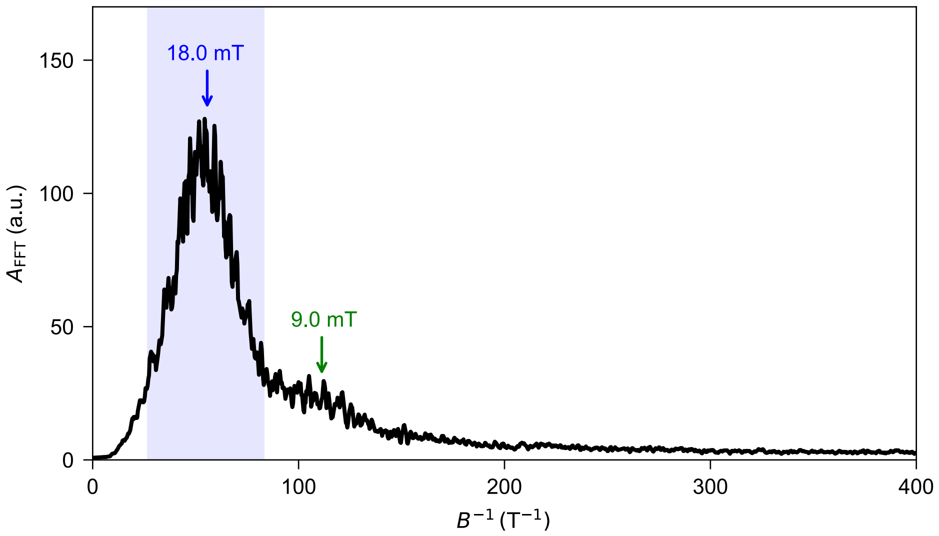

Supplementary Figure 3: Averaged Fourier spectrum of the magneto-conductance oscillations. Eight different magneto-conductance traces are measured and filtered with the Savitzky-Golay algorithm to remove slow oscillations such as universal conductance fluctuations. The Fourier transforms are then calculated and averaged to obtain the trace in the figure. The light blue window identifies the width of the spectrum expected from the area enclosed by the loop. The peak maximum at $55\,T^{-1}$ corresponds to the Aharonov-Bohm periodicity of $18\,mT$ and perfectly matches the expected value considering the area enclosed by the centre of the interconnected nanowires. A smaller, but discernible peak at $110\,T^{-1}$ indicates the presence of Altshuler-Aronov-Spivak oscillations.

Supplementary Note 4. Additional data in the multi-path Aharonov-Bohm regime

Two methods were used to study the phase evolution of the magneto-conductance oscillations for the device discussed in the main text. The data shown in Figs. 4 and 5 are taken by sweeping V_{PG} as the fast axis and $B_{\perp}$ as the slow axis. Oppositely, in Supplementary Figure 4 we present data taken with $B_{\perp}$ and V_{PG} being the fast and slow axis, respectively. The data presented here are taken at $V_{\mathrm{BG}} = 1.5\,\mathrm{V}$ as in Fig. 4 of the main text. For each value of V_{PG} (proportional to E_{QD}) the maxima of the AB oscillations are tracked, and their positions in $B_{\perp}$ are converted into the transmission phase φ via the AB periodicity and plotted in Supplementary Figure 4a. In Supplementary Figure 4b, we show the corresponding conductance trace (blue data points) exhibiting CPs. The phase displays a rapid evolution close to the charge degeneracy points. At the two inner CPs, the phase φ evolves by $\sim \pi$, and gradually shifts back to the original value. For the first and fourth CPs, the variation is much smaller than π. For all displayed Coulomb valleys, the phase is neither constant, nor does it exhibit a rapid phase lapse. Instead, gradual variations are observed, compatible with the picture discussed in the main text in which multiple trajectories with different enclosed areas in the nanowire interferometer couple to different QD orbitals.

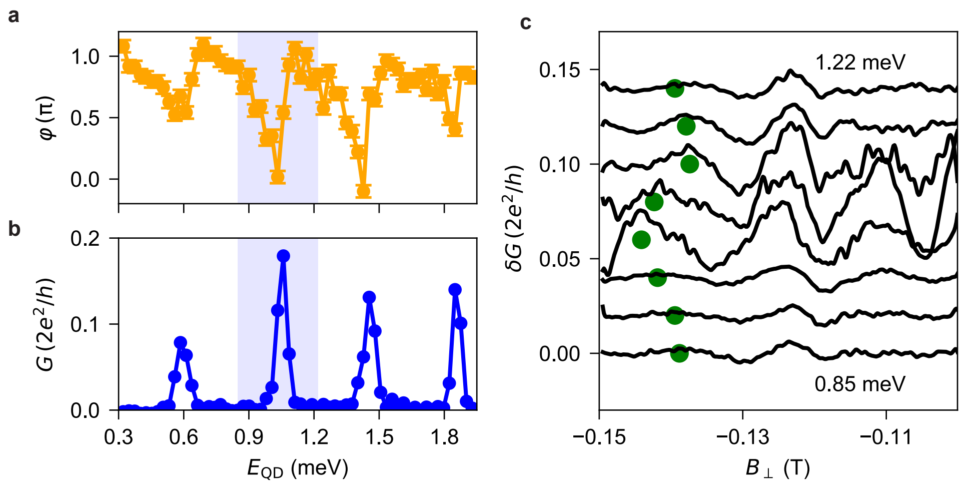

Supplementary Figure 4: Additional data in the multi-path Aharonov-Bohm regime. **a** Phase φ of the magneto-conductance oscillations as a function of E_{QD}, **b** G vs. E_{QD} exhibiting four CPs. **c** Aharonov-Bohm oscillations measured in the proximity of the second CP. δG is obtained by subtracting a slowly varying background from the magneto-conductance traces. The green circles identify the maxima of the oscillations in the first period. The traces are displaced for clarity.

Supplementary Note 5. Phase variations and fundamental symmetries

The top panel of Fig. 4d in the main text shows that the transmission phase swings from 0 to π at resonance over an energy range of $\sim \Gamma$. This continuous variation differs significantly from what has been reported in the pioneering experiment of ref. 1 where the phase was mysteriously locked to 0 and π with abrupt switches in between. Later on, their finding was understood in term of fundamental symmetries.

Time-reversal symmetry imposes, in fact, the two-terminal conductance to be an even function of the magnetic field (the Casimir-Onsager relation: $G(B) = G(-B)$). In an Aharonov-Bohm interferometer, the conductance displays sinusoidal oscillations on top of a background, and the symmetry around zero field imposes on their phase to assume only two values: 0 or π. However, in our experiment we find the opposite: the phase variation at resonance is smooth and not abrupt. We associate this behaviour with the fact that time-reversal symmetry does not hold in our context due to the large magnetic fields applied.

Supplementary Note 6. Fitting results

A. Main text Fig. 3

We obtain the dashed lines in Fig. 3a by fitting each peak independently with the single-trajectory model Aharonov-Bohm interferometer. For this and other fits, we use $J = 1$ and $ka = 1.57 \sim \pi/2$ similarly to ref. 2. The phase of J_L is a fitting parameter that is not relevant and therefore not displayed. We report the other values in Supplementary Table 1 subdivided into the three characteristic regimes in Fig. 3a.

	**Regime**		
**Average parameter**	low t_{ref} (green trace)	medium t_{ref} (blue trace)	high t_{ref} (orange trace)
j_L (μeV)	31 ± 1	34 ± 6	14 ± 8
J_R (μeV)	140 ± 6	202 ± 17	206 ± 26
t_{ref}	$< 10^{-3}$	0.04 ± 0.01	0.28 ± 0.02

Supplementary Table 1: Average and standard deviations of the best-fit parameters of four peaks in the three different regimes in Fig. 3a of the main text.

B. Main text Fig. 4

We obtain Fig. 4b by fitting the data of Fig. 4a with the single-trajectory model Aharonov-Bohm interferometer with $N = 2$. The fitting procedure is facilitated by fixing two parameters globally (i.e., for the entire measurement): a constant offset to the traces of $0.02 \cdot 2e^2/h$ and $t_{ref} = 0.07$. The phase of J_L is an independent parameter that increases with the magnetic field along the y-axis. We display in Supplementary Table 2 the best-fit parameters averaged along the magnetic field axis. Peak numbers are ordered from left to right.

	**Peak number**	
**Average parameter**	1	2
j_L (μeV)	192 ± 22	182 ± 12
J_R (μeV)	57 ± 10	59 ± 5
E_n (meV)	0.241 ± 0.001	0.618 ± 0.011

Supplementary Table 2: Best-fit parameters averaged along the magnetic field axis of Fig. 4. The errors presented are the standard deviations of the distributions of the parameters.

C. Main text Fig. 5

In order to obtain Fig. 5b of the main text, we first consider the line-cut of the data at $B_\perp = 0.667\,\mathrm{T}$ and fit the peaks independently with the single-path interferometer model. We fix some of the parameters to simplify the procedure as $t_{ref} = 0.05$ and the added offset to the trace at $0.06 \cdot 2e^2/h$. In this procedure, the phase of J_L is used as a free parameter and not relevant here. The other values are shown in Supplementary Table 3, where the peak names in Fig. 5a are ordered numerically from left to right.
We then extrapolate the trace with the best-fit parameters by varying the phase of J_L and adjusting the values of $x(n) \in [0.025,\ 0.01,\ 0,\ -0.016]$ to qualitatively reproduce the experimental data.

	**Peak number**			
**Parameter**	1	2	3	4
j_L (μeV)	178 ± 2	165 ± 4	153 ± 5	167 ± 2
J_R (μeV)	33.4 ± 0.2	60.9 ± 0.1	51.4 ± 1.1	37.3 ± 0.04
E_n (meV)	2.000 ± 0.001	2.293 ± 0.002	2.710 ± 0.002	3.092 ± 0.001

Supplementary Table 3: Fitting parameters obtained at $B_\perp = 0.667\,\mathrm{T}$ of Fig. 5a in the main text.

Supplementary Note 7. Cotunnelling Aharonov-Bohm effect in a second device

Here, we report additional measurements of Aharonov-Bohm interference in the cotunnelling regime for a second device. The electron density in this device (Supplementary Figure 5a) is tunable by voltages on a global back gate and several top gates. The top dielectric used here is Al_2O_3 grown via atomic layer deposition. A T-shaped quantum dot is formed in the bottom branch of the interferometer and has a charging energy of $E_c \sim 0.5\,\mathrm{meV}$, with the tunnel coupling to the leads adjustable using the tunnel gates T1 and T2. Although the conductance around $V_{SD} = 0$ is strongly suppressed owing to the Coulomb blockade (inset of Supplementary Figure 5b), the magneto-conductance exhibits clear Aharonov-Bohm oscillations with a periodicity of $9 - 11\,\mathrm{mT}$ (Supplementary Figure 5b, main panel). The oscillation amplitude is sizable despite the cotunnelling conductance being as low as $\sim 0.025 \cdot 2e^2/h$. This value corresponds to a typical dwell time in the QD in the order of $5 - 10\,\mathrm{ps}$. The magnitude of the Fast Fourier Transform (FFT) of these oscillations is displayed in Supplementary Figure 5c, together with the FFTs of the AB oscillations at gradually stronger cotunnelling conductance, until the Coulomb blockade is fully quenched (red trace corresponding to the 'open regime' with $G \sim 2e^2/h$). We observe here that the width of the FFT peak of the AB signal increases when quenching the Coulomb blockade by making the barriers more transparent (in particular, see the difference between the red and the other traces). This fact might suggest that, when the quantum dot is defined, fewer trajectories play a role in the transport through the interferometer, reducing the spread of the enclosed area.

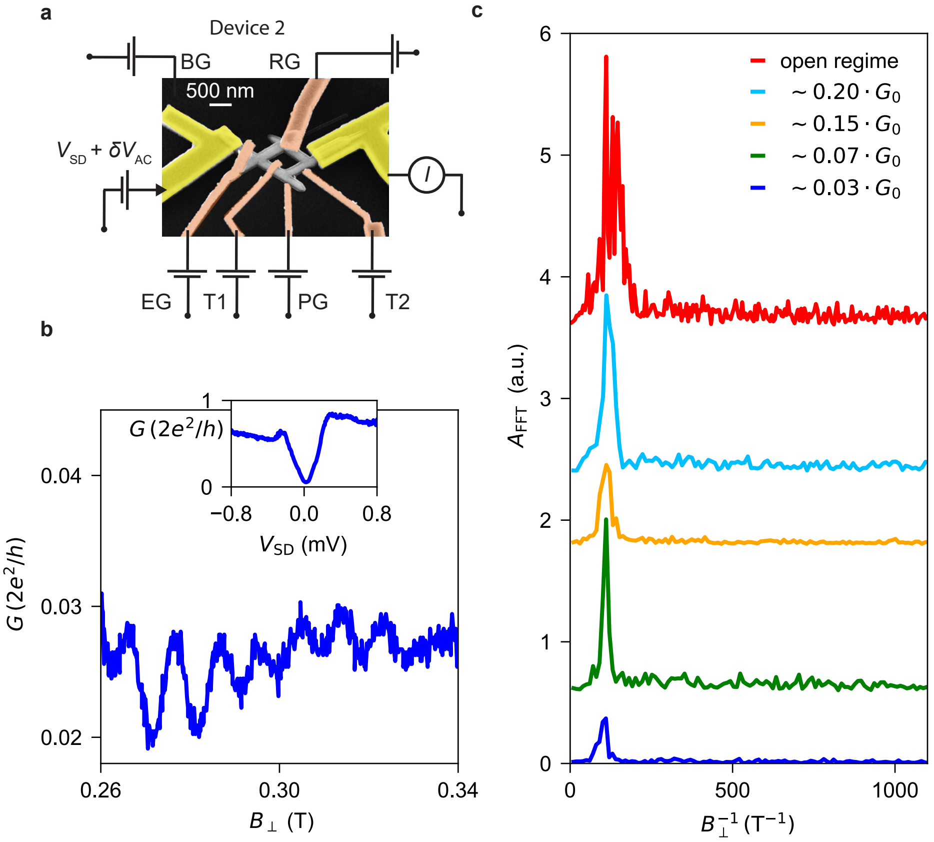

Supplementary Figure 5: Cotunnelling Aharonov-Bohm effect in a second device. **a** False-color scanning electron microscopy image of a second device. A quantum dot with a T-shape is formed in the bottom branch of the network by applying negative voltages to the two tunnel gates (T1 and T2). The induced charge is tuned via the plunger gate (PG). We use the top branch of the network as the reference arm of the interferometer, with a transmission tunable by the reference gate (RG). **b** Differential conductance G at zero bias voltage V_{SD} as a function of the perpendicular field $B_\perp$ in the cotunnelling regime manifesting AB oscillations with a period of $\sim 9 - 11\,\mathrm{mT}$. Inset: G vs. V_{SD} taken at $B_\perp = 0.34\,\mathrm{T}$. **c** Magnitude of the Fast Fourier Transforms for different tunnel gate settings. The legend indicates the average value of the cotunnelling conductance of the raw magneto-conductance traces.

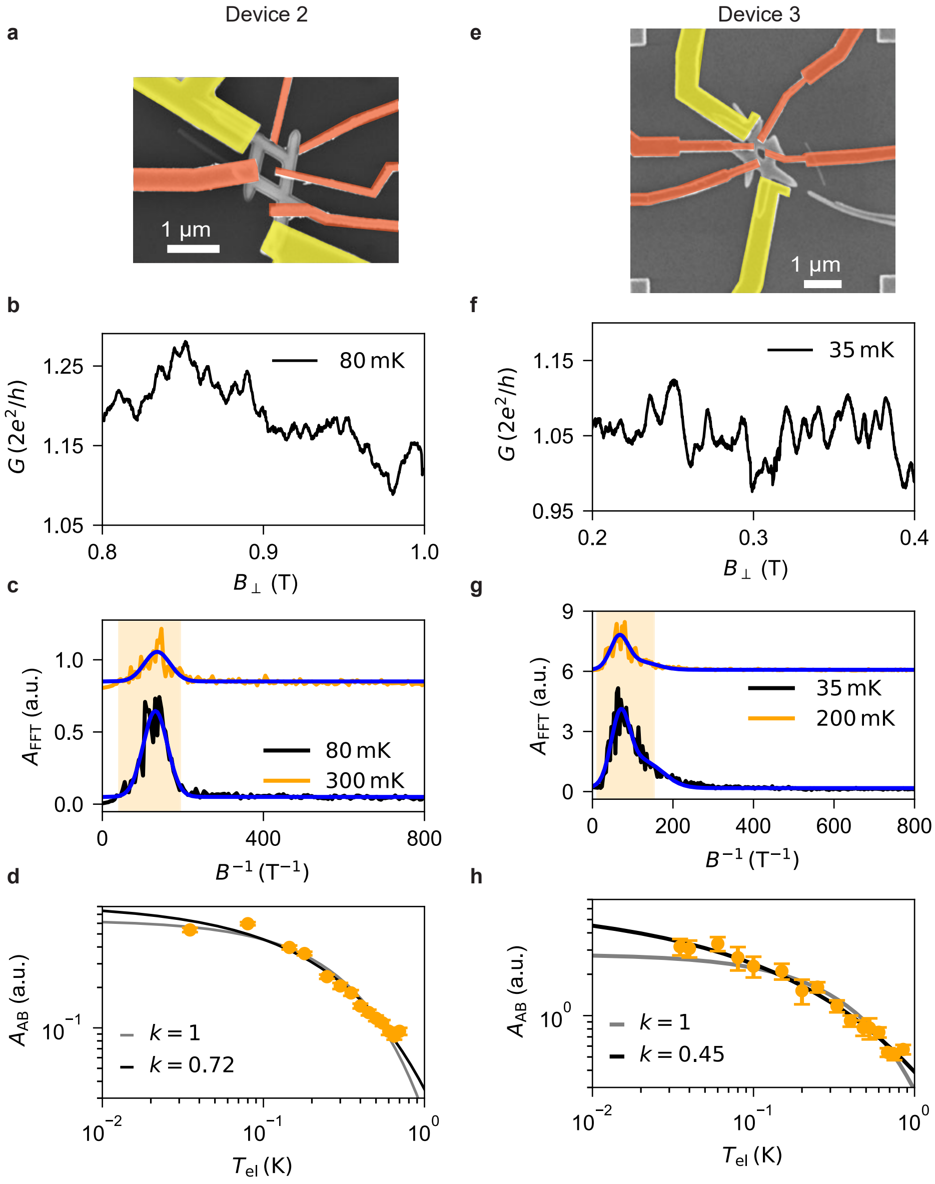

Supplementary Figure 6: Aharonov-Bohm temperature dependence of the second and third device. **a**, **e** Scanning electron micrographs of devices 2 and 3, respectively. **b**, **f** Typical magneto-conductance traces for the two devices exhibiting oscillations superimposed on a slowly varying background. **c**, **g** Average of several Fast Fourier Transforms of oscillations at two different temperatures fitted with a Gaussian in **c** and a sum of two Gaussians in **g**. The yellow shaded area denotes the expected range of the first harmonic (Aharonov-Bohm) according to the sizes of the loops. In **d**, **h** the temperature dependence of the AB amplitudes is presented for both the devices (orange points). Black and grey traces are the best fits of the data.

Supplementary Note 8. Temperature dependence of the Aharonov-Bohm effect

In Supplementary Figures 6a and 6e, we show the scanning electron micrographs of two nanowire loops (second and third devices) used to study the temperature dependence of the Aharonov-Bohm oscillations. In Supplementary Figures 6b and 6f, we plot two typical magneto-conductance traces of each device. They both exhibit AB oscillations superimposed on a slowly varying background. In the data analysis, we subtract this background and calculate the Fast Fourier Transform. The amplitudes A_{FFT} shown in Figs. 6c and 6g are the averaged Fast Fourier Transforms of several scans taken in the same magnetic field window. The peaks in the spectra are fitted with a Gaussian obtaining

the power spectrum of the AB oscillations A_{AB}. For the third device, we find that the sum of two Gaussian curves better describes the broad FFT peak due to the presence of the second harmonic. In panels d and h, we demonstrate the decrease of A_{AB} as a function of temperature. The amplitudes of each harmonic (with index n) are expected to decrease as $\exp(-n \cdot L/l_\phi)$, with l_ϕ being the phase coherence length and L being the effectively travelled path length [3]. In ballistic systems, we have $l_\phi \propto T^{-1}$, while $l_\phi \propto T^{-1/2}$ in the diffusive regime [4]. We fitted the experimental decay of the Aharonov-Bohm amplitude with the function $A \cdot \exp(-\alpha T^k)$, with free parameters A, α, k, and the Aharonov-Bohm phase coherence length defined as $l_\phi = (L/\alpha)T^{-k}$. The best-fit curves are shown in black, and correspond to $k_{\mathrm{best}} = 0.72$ and $k_{\mathrm{best}} = 0.45$ for the second and third devices, respectively. For comparison, we also show the best-fit curves obtained for $k = 1$. The values of the fitted exponents k reflect the nature of the transport in the semiconducting loops, which is in the crossover between completely ballistic and diffusive. In fact, the typically travelled length is $\sim 1\,\mu\mathrm{m}$, which is only a few times larger than the estimated mean free path of $\sim 0.1 - 0.3\,\mu\mathrm{m}$ [5, 6]. We expect that at higher temperatures and for the longer loops (cf. device 2) the diffusive model with $k = 0.5$ captures the experimental trend better than the ballistic one, but the experimental data do not provide conclusive evidence. From our analysis, we derive $l_\phi(35\,\mathrm{mK}) = \frac{L}{\alpha}T^{-k} \sim 8\,\mu\mathrm{m}$ for the second device, and $l_\phi(35\,\mathrm{mK}) \sim 2.5\,\mu\mathrm{m}$ for the third loop. It can be argued that these values are probably underestimating the actual coherence length since the amplitude of the AB oscillations (more generally all odd harmonics) are additionally suppressed by the energy averaging effect.

Supplementary References

[1] Yacoby, A., Heiblum, M., Mahalu, D. & Shtrikman, H. Coherence and phase sensitive measurements in a quantum dot. *Phys. Rev. Lett.* **74**, 4047–4050 (1995).
[2] Aharony, A., Entin-Wohlman, O. & Imry, Y. Phase measurements in open and closed Aharonov-Bohm interferometers. *Phys. E* **29**, 283 – 288 (2005).
[3] Ihn, T. *Semiconductor Nanostructures* (Oxford University Press, 2010).
[4] Ludwig, T. & Mirlin, A. D. Interaction-induced dephasing of Aharonov-Bohm oscillations. *Phys. Rev. B* **69**, 193306 (2004).
[5] van Weperen, I. *et al.* Spin-orbit interaction in InSb nanowires. *Phys. Rev. B* **91**, 201413 (2015).
[6] Ö. Gül *et al.* Towards high mobility InSb nanowire devices. *Nanotechnology* **26**, 215202 (2015).
